# Results of Treatment of Grades IV and V Vesicoureteral Reflux with Endoscopic Injection of Polyacrylate Polyalcohol Copolymer

**DOI:** 10.3389/fped.2013.00032

**Published:** 2013-10-28

**Authors:** Francisco Ignacio De Badiola, Ricardo Soria, Roberto Luis Vagni, María Nieves Ormaechea, Juan Manuel Moldes, César Benmaor

**Affiliations:** ^1^Hospital Italiano de Buenos Aires, Buenos Aires, Argentina

**Keywords:** endoscopic injection, high grade vesico-ureteral reflux, vantris, polyacrylate polyalcohol copolymer, children

## Abstract

**Purpose:** Here we report the results of a review of a prospectively maintained database of the use polyacrylate polyalcohol copolymer (PPC) injection to correct grades IV and V VUR.

**Materials and Methods:** All children with grades IV and V primary VUR that presented with febrile urinary tract infection while on prophylaxis, in a 3-year period, were treated with a sub-ureteral injection of PPC. Institutional ethical approval was obtained. Exclusion criteria were incomplete bladder emptying documented on videourodynamic study, ureteral duplication, paraureteral diverticula, and poor ureteral emptying observed during fluoroscopy and previous open surgical or endoscopic treatment. Pre- and post-operative evaluation included urinalysis, renal and bladder ultrasonography, DMSA scan, and videourodynamic studies.

**Results:** Thirty-three children [36 renal units (RU)] were included with a median age of 57 months (range 7–108). There were 18 boys and 15 girls. Thirty RU had grade IV and 6 grade V VUR. Median follow-up time was 32 months (range 7–58). Reflux was cured in 32/36 RU with the first injection, but another two patients were reimplanted because of dilatation. Complications included early urinary tract infection in seven children, transient lower urinary tract symptoms in five children. Progressive ureteral dilatation was noted in four children and was treated with insertion of a double J stent. Two of these children eventually required an ureteroneocystostomy.

**Conclusion:** The use of PPC to treat grades IV and V vesicoureteral reflux in young children has an overall success rate of 83.3%. Persistent ureteral dilatation was present in 11% associated with high injection volume. Future studies will attempt to maintain a high success rate reducing the volume of injection and the incidence of dilatation.

## Introduction

The treatment of grades IV and V primary vesicoureteral reflux (HGVUR) is dependent on the age of the child, symptoms, and status of the kidneys. Given the high-resolution rate of reflux in children, medical treatment with antibiotic prophylaxis is preferred (1). However, the resolution rate of HGVUR is low (1). When surgical treatment is required because of non-resolution, breakthrough infections, or low expectation of spontaneous resolution, many urologists choose the endoscopic injection. There is a parental preference for endoscopic treatment. This carries an acceptable success rate with lower morbidity and cost, no scars, as an outpatient procedure (2, 3). The initial experience with polyacrylate polyalcohol copolymer (PPC) in a multicenter study in all grades of VUR had an 83.6% success rate (3). The possible benefit of PPC for treating HGVUR is the long term of the results since it is a non-absorbable substance.

Here we report the results with the use of a new substance, PPC (Vantris^®^-Promedon Argentina) in a single center study including only patients with primary HGVUR.

## Materials and Methods

All consecutive children presenting with febrile urinary tract infections (UTI) under prophylaxis and HGVUR in a 3-year period at one institution were included in this study. The patients were enrolled prospectively. Institutional ethical approval was obtained.

All patients had received antibacterial prophylaxis from 6 to 18 months, in 5 there was a prenatal history of urinary tract dilatation. Exclusion criteria were bladder and bowel dysfunction, ureteral duplication, paraureteral diverticula, poor ureteral emptying observed during fluoroscopy, and previous open surgical or endoscopic treatment. We considered good ureteral emptying when there was no residual contrast in the ureter at the end of voiding.

Preoperative evaluation consisted of urinalysis and culture, renal and bladder ultrasonography, DMSA scan, and videourodynamic studies. Post-operative studies included renal and bladder ultrasonography, urinalysis and culture 10 days, 3, 6, and 12 months after the procedure. DMSA scan and videourodynamic studies were repeated 1 year post-operatively.

Reflux was graded according to the international grading system, based on the voiding cystourethrogram performed at the time of the videourodynamic studies.

Success was defined as the elimination of HGVUR with a single injection.

Complications were classified as UTI, progressive ureteral dilatation, new renal damage, or persistent reflux. Decreases in the differential renal function of more than 5% or the appearance of new scars were considered evidence of new renal damage. Obstruction was defined as progressive ureteral dilatation of more than 5 mm, 3 months after the procedure.

Clinical data were collected in a prospective manner. Patient demographics, the presence lower urinary tract symptoms (LUTS), ureteral and upper tract dilatation, UTI, presence or absence of VUR at 12 months, and changes in DMSA scan were recorded.

All patients were injected with PPC. This is non-absorbable substance, with average particle size of 320 μm, causes minimal inflammatory reaction and it and does not migrate.

### Surgical technique

The endoscopic injection was performed under general anesthesia following established techniques. A 10 Ch cystoscope was used. The injection was performed manually with a 1 ml syringe provided by the manufacturer, through a 23G needle. We used the hydrodistention technique (4) and injected intra- and sub-ureteral until the ureteral walls coapted.

Patients were kept on antibacterial prophylaxis for 10 days after the procedure unless the first ultrasound showed ureteral dilatation.

## Results

Thirty-three patients were included [36 renal units (RU)]. Another 27 patients with HGVUR were operated in the same period but match the exclusion criteria. The median age was 57 months (range 7–108), 18 were males and 15 females. Thirty-six RU were treated. Of these 20 were on the right side, 10 on the left, and 3 bilateral. HGVUR was grade IV in 30 and grade V in 6 RU. Twenty-one of the RU had abnormalities on the DMSA scan preoperatively. The mean volume injected was 2 ml (range 1–3 ml). The median follow up period was 32 months (range 7–58 months).

### Elimination of reflux

Videourodynamic study at 1 year after the procedure revealed HGVUR resolution in 32 of 36 RU (88.9%) of the RU with persistent reflux, 4 had grade IV in the preoperative evaluation. The mean injected volume of PPC 2 ml (1–3 ml).

### Complications

Post-operative febrile UTI were observed in nine patients, 7 (21%) during first 2 weeks after surgery but they did not recur despite absence of antibacterial prophylaxis and 2 (6%) recurrent UTI associated to persistent VUR. Five children (15%) had transient LUTS as urgency-frequency managed with anticholinergics.

Ten patients (27%) had ureteral dilatation on the first ultrasound, but only in four the ureter stay dilated and in fact the progressive dilatation increased extending to the proximal urinary tract at the 3-month control. A double J stent was inserted in these four patients for a 4-month period. Two of these patients had a dilated ureter preoperatively. In two cases the dilatation resolved after removal of the stent. The other two patients underwent a unilateral extravesical ureteroneocystostomy for persistent dilatation after removal of the JJ stent. All dilated ureters had been injected with 2.5–3 ml of PPC.

DMSA scans were obtained at 1 year in 19 patients. In three cases changes were observed compared to the preoperative scan. One girl with persistent reflux and a febrile UTI developed a new scar. Another girl with persistent grade III reflux suffered a 7% reduction in differential function. Finally one asymptomatic boy with reflux resolution and without dilatation also suffered an 8% decrease in function on the involved side and was considered as a technical error.

The four patients (4 RU) with persistent reflux also underwent unilateral extravesical ureteroneocystostomy. The overall success rate was 83.3% including the persistent reflux and the two patients who require a reimplantation because of persistent ureteral dilatation.

## Discussion

The management of vesicoureteral reflux has been a matter of debate for decades. The potential advantages of surgical versus medical treatment with prolonged administration of antibacterial prophylaxis had not been defined yet (1). More recently, the role of antibiotic prophylaxis has been also questioned (5). These uncertainties have not prevented progress in the development of endoscopic injection of bulking substances to treat reflux (2).

Following the initial report by Matouschek in 1981 (6), O’Donnell and Puri (7) demonstrated the successful the use of ethylene polytetrafluoro paste (Teflon) injected sub-ureterally to control reflux. However, concerns regarding potential migration of the substance, the formation of granulomas, and lack of approval by the US Food and Drug Administration (FDA), hampered the wide spread application of this treatment outside Ireland and a few other countries (8).

Polydimethylsiloxane paste was shown not to migrate and was also effective to control reflux. Although it remains the subject of occasional reports, injection of this substance, which is very viscous, requires a special pressure syringe a method many find cumbersome. Again, failure to obtain FDA approval limited its use in the US (9).

The use of cross-linked bovine dermal collagen failed to obtain wide spread acceptance because of low success rate and durability and the need to perform sensitivity tests prior to the injection. The landscape of endoscopic injection for the treatment of reflux rapidly changed with the appearance of hyaluronan/dextranomer copolymer (Deflux^®^) after the acceptance by the FDA and in many centers became a first line of treatment for vesicoureteral reflux (10). Whereas some authors have reported excellent success for HGVUR with up to three injections (11), others have shown only 41% cure rate for grade IV reflux (12).

In 2008, we reported the development of a new agent design for endoscopic treatment of vesicoureteral reflux, namely PPC. The product is quite fluid and can be injected with and ordinary syringe through a 23 G needle (13).

The preliminary results from a multicenter study demonstrated an 83.6% elimination of reflux with a single injection. However in that series the majority of the patients had grades II and III reflux (3). In the present study we report the results of injection of PPC in grades IV and V reflux.

This study is unique in that all patients underwent a post-operative study 1 year after the injection to determine the presence of VUR. We chose to do videourodynamic studies pre and post-operatively since we have this capability readily available in our imaging department.

At the onset we decided to exclude patients with slow drainage of the upper tracts following reduction of voiding pressures emptying (poor ureteral emptying), since they may have an element of ureterovesical obstruction that may affect outcome.

We succeeded in curing HGVUR in 89% (32/36) of the ureters treated with a single injection, but two other patients needed to be reimplanted because of persistent ureteral dilatation with a final success rate of a 83%. This success rate is comparable as that reported in other series (11). Also, we did not consider patients with residual low grade reflux as cured. This is a small number of patients and further studies are needed to reach statistical significance.

The early US show a 27% transient ureteral dilation, mostly because of post-operative edema and UVJ obstruction.

Our rate of obstruction (at 3 months US) requiring treatment was 11% (4/36) and 5.6% (2/36) required open surgical treatment for persistent ureteral dilatation. Two of the four patients dilated had dilated ureter prior to the injection. Since the four patients had symptoms and progressive dilatation on ultrasound, we did not considered a renal scintigraphy necessary to confirm the obstruction.

Vandersteen et al. reported a 0.7% obstruction with dextranomer/hyaluronic acid copolymerin in 1,155 ureters treated injection, managed only with a stent (14). Mazzone et al reported the highest complication rate with the same substance in 5.7% of 87 ureters (15).

The reason a substance may fail could be because of the technique used (volume used and/or place of injection), inflammatory reaction or the substance itself. In these four patients with persistent ureteral dilatation the volume injected exceeded 2 ml.

The incidence of post-operative febrile UTI during first 2 weeks after surgery in this series (21%) (7/33) was comparable to that reported by Sedberry-Ross et al (16). In their series, early febrile UTIs were predictive of treatment failure even in the absence of reflux in an early voiding cystourethrogram. Longer follow up is needed to determine if this will also be the case with PPC. The 15% (5/33) incidence of LUTS in this series was transient and unrelated to either infection or obstructive complications.

The weakness of this study is the small number of patients and the strengths is the long-term follow up of a non-absorbable substance.

In summary, our experience with the use of PPC to treat primary HGVUR has been favorable. Since some reports suggest that results with the use of Deflux^®^ may not be durable (17), the use of a non-biodegradable substance with minimum inflammatory reaction such as PPC may obviate this shortcoming and risk of obstruction of endoscopic treatment. To test this hypothesis we plan long-term follow up of our patients. Future protocols will attempt to reduce the volume of substance injected and expect to maintain the high success rate with a single injection.

## Conflict of Interest Statement

Dr Francisco de Badiola serves as a consultant for Promedon Argentina.
